# The oscillatory response of the electroretinogram and neuronal adaptation

**DOI:** 10.1111/aos.70105

**Published:** 2026-02-18

**Authors:** Lillemor Wachtmeister, Anders Eklund

**Affiliations:** ^1^ Department of Clinical Sciences/Ophthalmology Umeå University Umeå Sweden; ^2^ Department of Diagnostics and Intervention/Biomedical Engineering and Radiation Physics Umeå University Umeå Sweden

**Keywords:** electroretinogram, neuronal adaptation, oscillatory potentials, retinal diseases

## Abstract

After more than 50 years, there still remains a challenge and an interest to know more as well as extend and deepen our understanding of the small rapid wavelets, the oscillatory potentials (OPs), of the electroretinogram (ERG) and the neuronal adaptation of the retina. This paper summarizes the current knowledge of the rapid oscillatory response and its role in the process of neuronal adaptation in the retina. An effort has been made to enhance our ability and possibility, especially in the clinic, to interpret and use this response. The first section in short presents historic aspects, definitions and understanding of neuronal adaptation and the OPs. The second part deals with different and modern techniques and possibilities to record and measure the OPs, ffERG (full‐field ERG), mfERG (multifocal ERG), fmERG (focal macular ERG) and optimal adaptational recording conditions. The third section overviews the current apprehension of the origin(s) of the OPs. The fourth part describes the normal development of the OPs from prematurity to old age. The last section enlightens the importance of the OPs as indicators of disturbed retinal, neuronal adaptive function in different diseases in the retina.

## 1 INTRODUCTION

### 1.1 Historic aspects and definitions

#### 1.1.1 Neuronal/network adaptation

Two different systems of adaptation operate in the retina when the eye adapts for seeing in light and darkness, the photochemical adaptation and the neuronal adaptational one. The photochemical process is well known, a very slow working system that is related to the concentration of photopigments in the photoreceptors in the retina. The human eye will need about 30 min to adapt for seeing in the dark.

Bleaching and regeneration of photopigments was first discovered (in the frog) at the end of the 19th century and described by Boll (1876) and a couple of years later by Kuhne (1878). More than 50 years afterwards, Wald (1938) and Hecht & Wald (1933) could identify the substance as rhodopsin and the importance of Vitamin A in vision. About 30 years later, Dowling (1960a, 1960b) quantitatively established that the photochemical adaptation is indeed characterized by bleaching and regeneration of photopigments (rhodopsin).

Dowling (1963) and Dowling & Hubbard (1963) identified and described the two processes of adaptation and the differences between them. In strong background light intensities that bleached measurable concentrations of rhodopsin, there was a log‐linear relationship between visual sensitivity measured electrophysiologically and the content of rhodopsin in the rod‐dominated retina (of the rat). However, in weak background light that bleached non‐measurable concentrations of rhodopsin, there was found not to be such a relationship. In such case, the retina also rapidly regained its visual sensitivity in darkness. This adaptation process—fast and not depending on the content of visual pigments in the retina—implying on a swiftly working system and relying on neuronal activity and/or a network of neurons was consequently termed ‘network’ or neuronal adaptation.

To summarize in short to define, differentiate between the two systems of adaptation and describe neuronal adaptation and its relation to the photochemical adaptation:

1. In dim background light, the visual threshold is set by a network of neurons in the retina.
2. In bright background light, the photoreceptors determine the visual sensitivity to light of the retina.
3. During dark adaptation immediately after exposure to a strong background light, the very early rapid phase of recovery of visual sensitivity to light is dependent on the network of neurons in the retina. During the tardier, slower phase of dark adaptation to follow, the photoreceptors decide the threshold of the retina to discern light.

#### 1.1.2 Scotopic, mesopic and photopic adaptational conditions

Since the 1950s, the change of sensitivity of the photoreceptors (rods and cones) with time has been measured psychophysically after exposure to a bright background light in a Goldmannn–Weekers adaptometer and presented in an adaptometric curve (Dieterle & Gordon, 1956). As well known the progress of cone readaptation to darkness is comparatively fast, which is indicated by the cone link part of the adaptometric curve, whereas the sensitivity of the rods has a slower return, the rod link of the curve. The transition zone between the cone and rod links, the rod/cone break (Kohlrausch kink), indicates that the sensitivity to light is determined by both the cones and the rods.

To summarize concisely, the ‘cone link’ represents *photopic adaptational conditions* at which the adaptational state, the sensitivity of the retina, is determined by the cones. Second, the ‘rod link’ reflects *scotopic adaptational conditions* at which retinal light sensitivity is set by the rods. The rod–cone transition zone at which the sensitivity of the eye is regulated by both the rods and cones constitutes *mesopic adaptational conditions*. Scotopic background illumination has been found to correspond to the intensities of illumination below that of the cone threshold (Le Grand, 1968; Walters & Wright, 1943) and the mesopic level to illumination above cone threshold but below the level of rod saturation (Aguilar & Stiles, 1954; Rushton, 1961), that is, from 10^−2^ to 10^0^ cd/m^2^.

### 1.2 The oscillatory potentials (OPs) of the ERG

Since the first recording of the field potential of the retina in response to light, the electroretinogram, ERG several methods of recording have evolved (Holmgren, 1865). The most frequently used and that contain oscillatory potentials (OPs) are the full‐field ERG (ffERG), multifocal ERG (mfERG) and focal macular ERG (fmERG) and will be presented in the following part.

#### 1.2.1 ffERG

**FIGURE 1:**
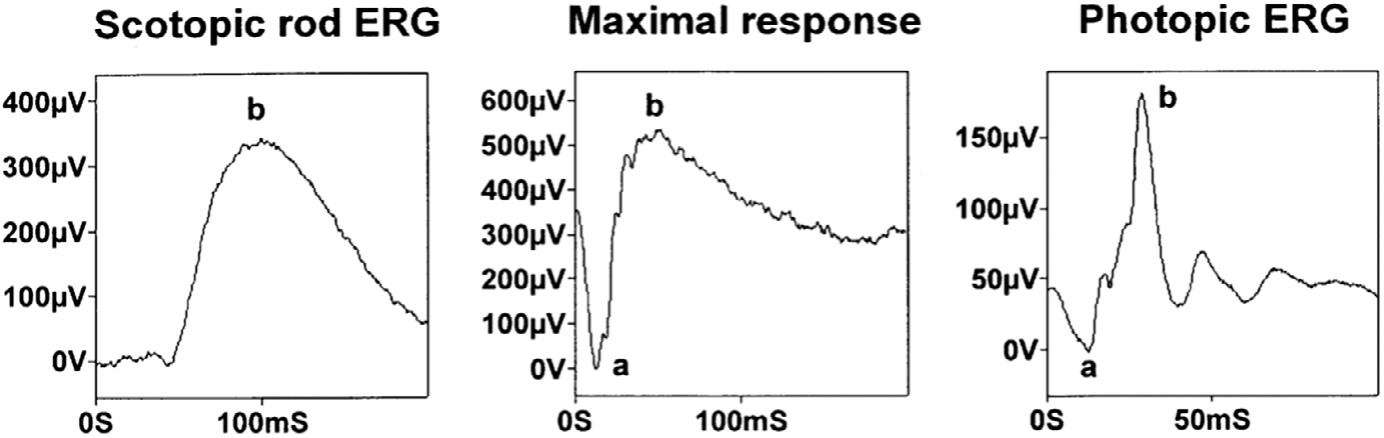
Normal ffERGs showing scotopic rod ERG, maximal response ERG and photopic ERG. Reproduced with permission from Holder (2001), Progress in Retinal and Eye Research, © Elsevier.

The conventional *ffERG* is a long extended electrical response and is a mass potential integrated from the whole retinal area and its different layers. It contains several and various components. In the clinic, usually a limited part of the ffERG is analysed, the major components, the a‐ and b‐wave (Figure 1).

The scotopic, rod‐driven ERG assessing the activity of the human rod photoreceptors (and bipolar cells) and the photopic, cone‐driven ERG being spatially heterogeneous and a mix of responses from different retinal regions are both valuable clinical tools to provide us insights into the function of the normal retina as well as in disease.

**FIGURE 2:**
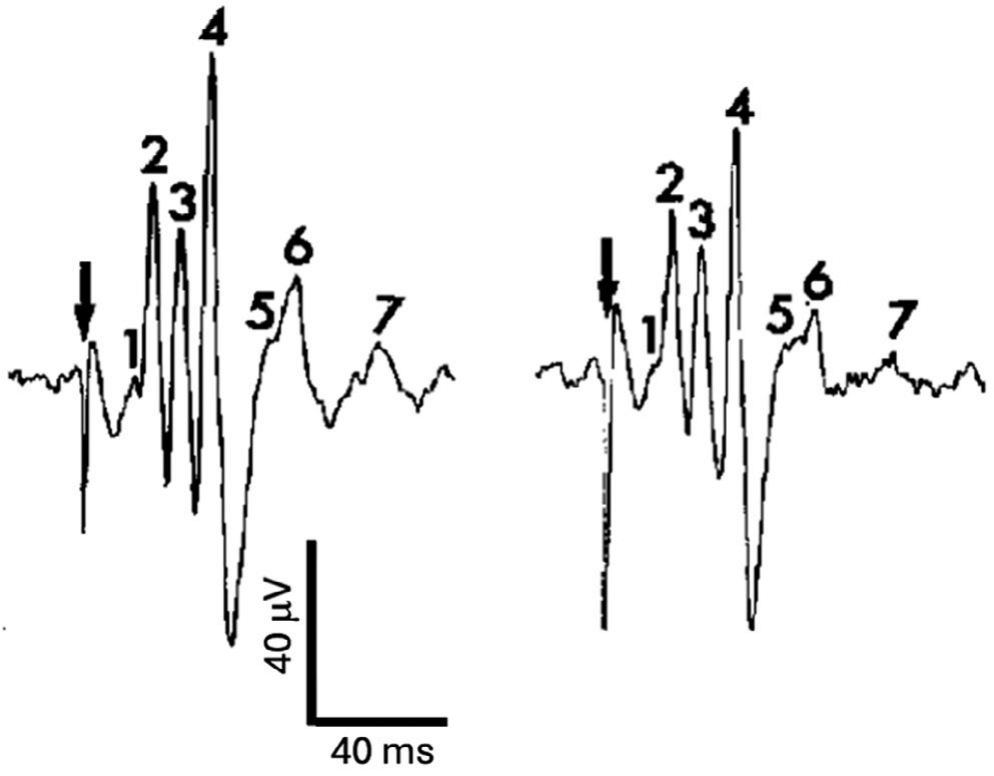
ffERGs showing photopic OPs (OP1–OP7) from two normal persons. Reproduced with permission from Lachapelle (1990) Documenta Ophthalmologica, © Springer.

The photopic ERG contains contributions from the retinal cone system, on and off bipolars. There are also high‐frequency components, the OPs. The OPs are small wavelets superimposed on the b‐wave of the ffERG and the main subject of the present survey (Figure 2).

**FIGURE 3:**
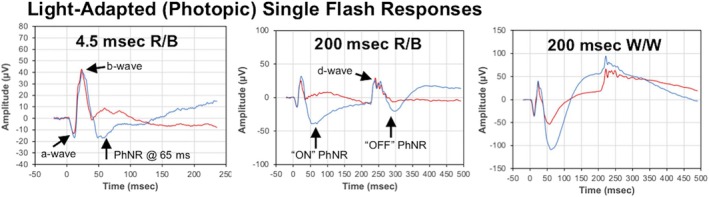
Normal light‐adapted (photopic) single flash ffERG responses showing oscillatory potentials (OPs) on both ON and OFF responses. These are most prominent in the 200 ms white‐on‐white (W/W) recordings, but also visible in the 200 ms red‐on‐blue (R/B) recordings, particularly on the OFF response. Blue trace corresponds to controls and red to glaucoma patients. Reproduced with permission from Dunn et al. (2023), Translational Vision Science & Technology, (Fig 1 Panel A) © Dunn et al, CC‐BY‐NC‐ND 4.0.

Under special stimulation conditions, the photopic ffERG contains separate and distinct ON and OFF responses. The ON component is considered to be related to the b‐wave and the OFF component to be associated with the d‐wave. Both components have been shown to contain wavelets (Dunn et al., 2023) (Figure 3).

There are also other components of the ffERG like the flicker response, early receptor potential (ERP), photopic negative response (PhNR), scotopic threshold response (STR) and others. They are of interest, studied and analysed in clinical as well as in experimental laboratories. To the author's knowledge, these other components of the ffERG do not contain wavelets like the OPs, and thus, these responses will remain beyond the scope of this survey.

#### 1.2.2 mfERG

**FIGURE 4:**
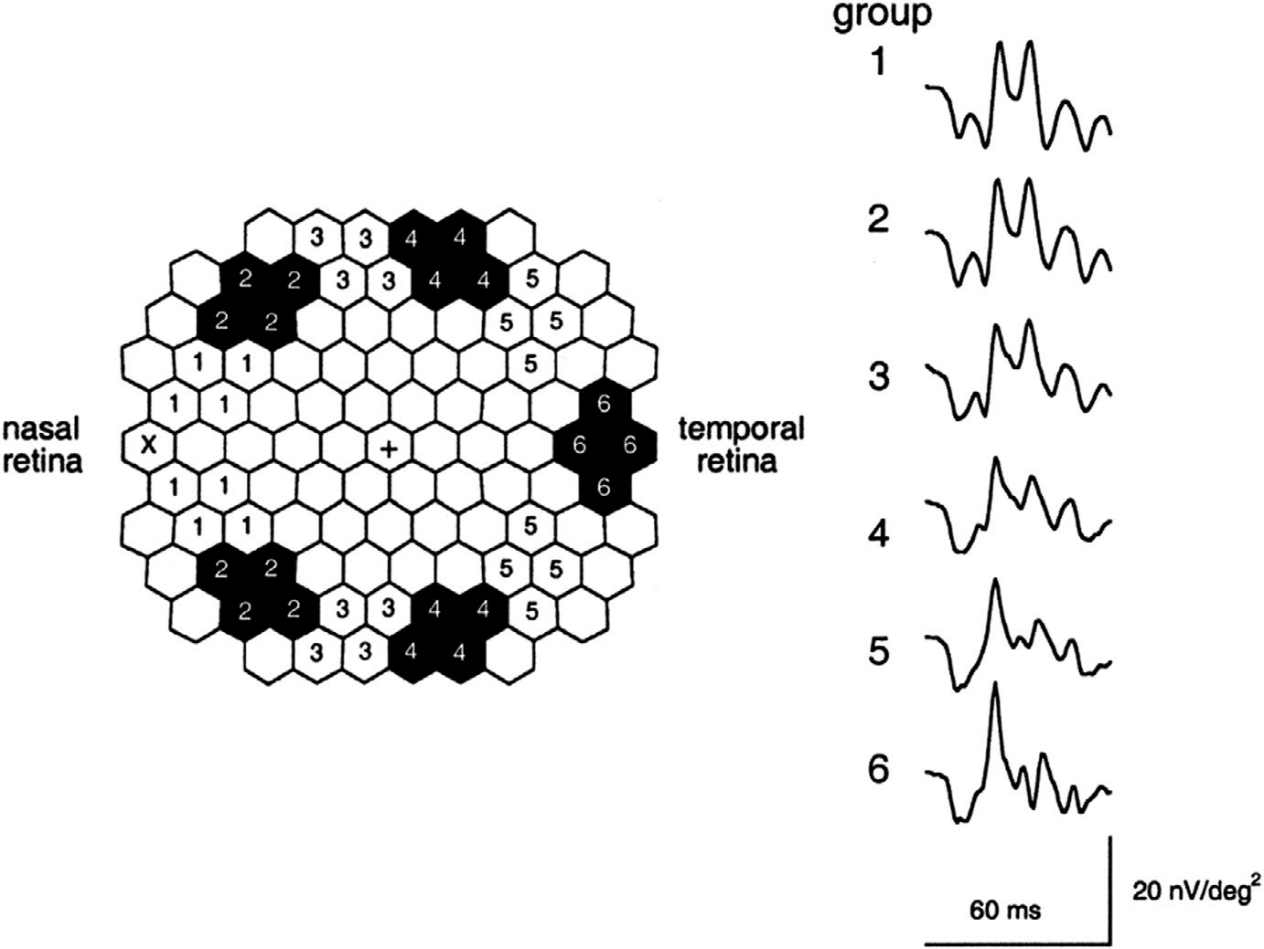
Normal mfERGs showing ‘little ERGs’ which consist of the same elements as the positive waves (b‐waves and OPs). Temporal/nasal variations are also observed. Reproduced with permission from Hood, 2000, Progress in Retinal and Eye Research, © Elsevier.

In the 90th a new technique was developed, the cone‐driven, *multifocal ERG* (mfERG) by Sutter and colleagues (Sutter, 1991; Sutter and Tran, 1992), which is widely used. Depending on the sequence of stimulation, a multifocal ERG will appear as ‘little ERGs’, reasonably close to responses obtained with more traditional focal stimulation (Miyake, 1990). One component, P1, is comprised of the same components as the positive waves (b‐waves and OPs) (Figure 4).

Fonseca et al. (2022) studying the relation of the photopic mfERG (N1, P1 and N2) to different layers of the human retina also found late appearing conspicuous oscillations in the overall mfERG. The stimulus display used in mfERG‐recording subtends usually 21–25° up to 43° in retinal radius. Thus, this response mainly and largely represents the function of the central part of the posterior pole of the eye.

#### 1.2.3 fmERG

**FIGURE 5:**
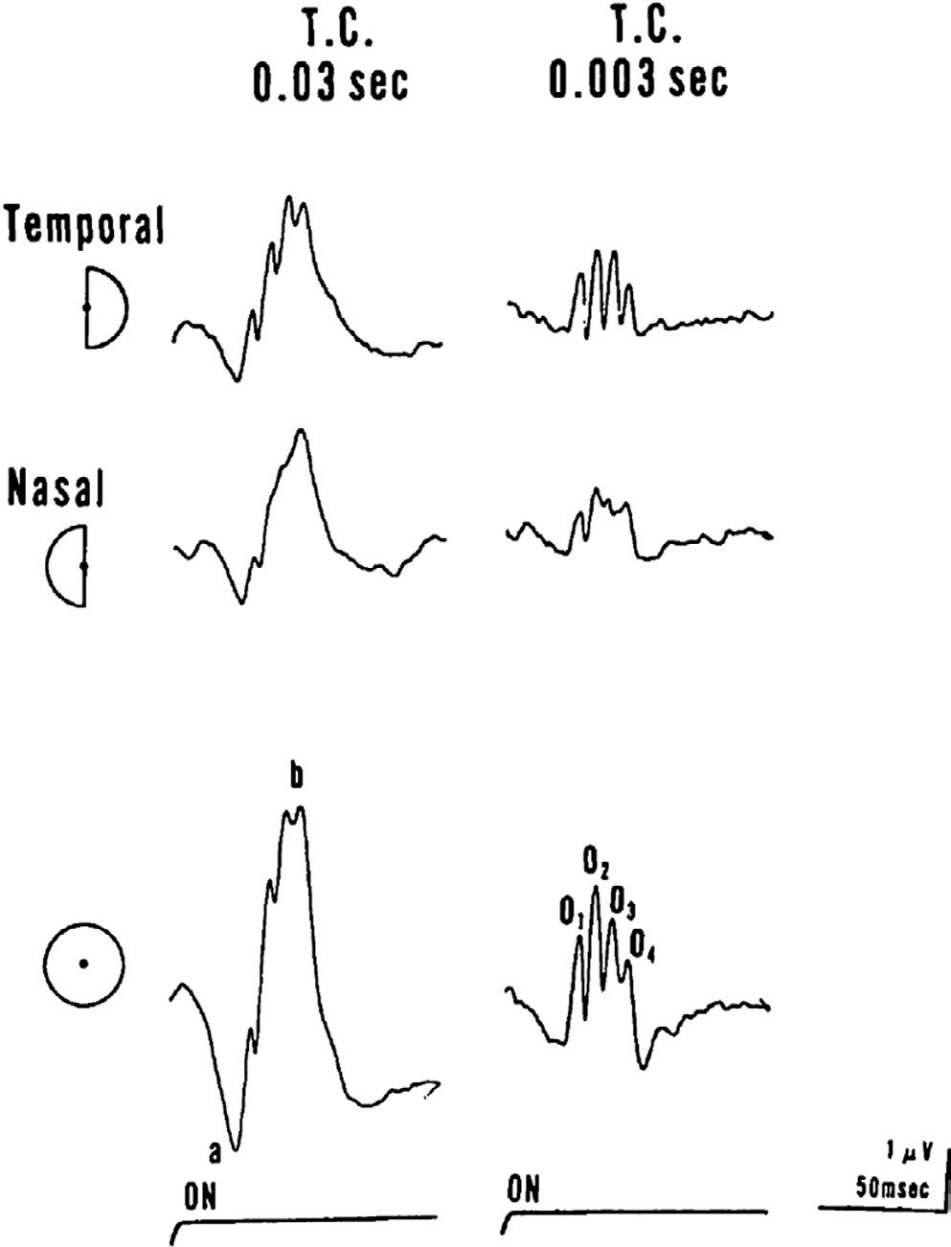
fmERGs from one normal person showing a pronounced asymmetry in the oscillatory response, with significantly larger amplitudes in the temporal retina compared to the nasal retina. Reproduced with permission from Miyake et al. (1989), Invest Ophthalmol Vis Sci, © ARVO.

The *focal macular ERG* (fmERG) is also mainly generated by the cone system in the macular region, elicited by spot stimulus, centred at the foveola and ranging from 5 to 15° visual angle either as a spot, hemicircular or a ring (Miyake et al., 1989). fmERG presents large three to four wavelets and shows spatial asymmetry being much larger when recorded from the temporal part of the macula (Figure 5).

Another electrophysiological response obtained from the central part, the macular area, is the *pattern ERG* (PERG). In a clinical setting, a pattern stimulation (of about 16 × 13 degrees visual angle) consisted of a black and white checkerboard or grating is used. It enables a direct measurement of ganglion cell function provided that the photoreceptor(s) function normally (Holder, 2001). Depending on temporal, spatial tuning and contrast, it is composed of three major components, N35, P50 and N95. These may have different and separate origins and P50 may in part have its origin distal to the ganglion cells and reflect a contribution from cone ON bipolar cells. It is a valuable complement in the electrophysiological protocol and practice. However, to the authors' knowledge, no oscillatory components in the PERG have been observed and reported.

## 2 TECHNIQUES OF RECORDING AND ANALYSING THE OPS

### 2.1 Full‐field, multifocal and focal macular ERG techniques

There is a range of techniques to record the ERG as an essential tool for clinical diagnosis and visual research. This part will briefly outline the main three techniques currently in use to record the oscillatory response of the ffERG, mfERG and fmERG, respectively.

#### 2.1.1 ffERG

The *ffERGs* of all species hitherto studied have been found to contain OPs. For example, the rod‐dominated mice retina (Watanabe & Hellner, 1974), the cone‐rich retina of the turtle (Armington, 1954) and the mixed rod–cone retina of the zebrafish all express an oscillatory response (Nadolski et al., 2021).

The OPs may be difficult to discern. Recording techniques and protocols are of great importance for the outcome of the results of a study and analysis to follow. Several recording methods and ways to isolate the OPs are used to obtain optimal, qualitative, quantitative and reliable measurements.

Since 1972 (Algvere and Wachtmeister, 1972), it is known from Fourier analysis that the OPs of the ffERG in man have a much higher dominant frequency (90–160 Hz) than the a‐ and b‐waves (about 25 Hz). Therefore, a passive (24 db/octave) bandpass filter (e.g. 80–500 Hz), i.e. a short time constant, may preferably be used to reduce the slow potentials, a‐ and b‐waves. This method makes the individual oscillatory peaks more conspicuous and clear to observe for measurements of amplitudes and implicit times, as well as for evaluation of the energy of the oscillatory response. In clinical practice, a digital filtering between 75–80 and 160–300 Hz (−3 db points) is often used to obtain the most easily visible OPs.

However, in experimental studies, other pass band settings have to be used and adjusted to choice of species, maturation and age. In mice, for example, the cone‐driven OPs have a lower dominant frequency (70–90 Hz), and the rod‐driven ones a higher frequency (100–120 Hz) and that the cone‐driven power only contribute to about 5% of the total power of this rod‐dominated retina (Lei et al., 2006).

Other methods and protocols are in practice to record, filter and analyse the OPs of the ffERG. For example, Fulton et al. (Akula et al., 2007) used a discrete Fourier transform (DFT) to evaluate the OPs' dominant frequency, power spectrum and sensitivity of the dark‐adapted ffERG. Short flashes delivered between 2 s and 2 min were used. The a‐wave (P3) was digitally subtracted, and a second‐order Butterworth filter (60–235 Hz) was applied. The post‐receptor (P2) single non‐averaged responses were analysed. The signal‐to‐noise ratio was found to be low and especially advantageous. In the frequency domain, the spectra were bimodally distributed with peaks at about 100 Hz and about 140 Hz.

Recently, focus has been on studying the OPs of the ffERG in response to dim flashes and the importance of measuring the implicit times (Kim et al., 2018; Pardue et al., 2014). For example, in mice, scotopic OPs were obtained using an interstimulus interval from 4 to 65 s, and data analysis was performed after extracting the OPs using a bandpass filter setting of 75–500 Hz (Kim et al., 2018). Furthermore, rod‐dominant dim luminance responses were compared to bright flashes, to compare rod pathways versus mixed rod‐cone pathways respectively, both in rodents (mice and rats) as well in humans (Pardue et al., 2014). Tentatively, the scotopic OPs may plausibly and potentially serve for an earlier detection of retinal dysfunction than a mixed rod/cone response.

Lachapelle and co‐workers (Gauthier et al., 2019; Lachapelle, 1990) using averaging technique and steady background illumination quite recently evaluated various filtering techniques of the photopic OPs of the ffERG. Gauthier et al. (2019) found a bimodal band in the photopic OPs, at 70–80 Hz and at 130–160 Hz, respectively, and the Butterworth filter most faithfully reproduces the high‐frequency photopic OPs.

Hancock and Kraft (2008) used a filter setting of 120–180 Hz to measure the sum of time to peak (TTP), the sum of amplitudes (OP1–OP4), the area and power around 150 Hz of the dark‐adapted averaged oscillatory response of the ffERG in humans (*n* = 15). Brighter stimuli up to 40 cds/m^2^ were used. The timing of the OPs was described to accelerate, but the amplitudes, area and power were described to decline with higher stimulus intensities.

A discrete wavelet transform (DWT) method has been described by Gauvin et al. (2015) to analyse the oscillatory component of the photopic ffERG. An attempt to specifically associate their function to ON and OFF components was made and may be useful in the future.

Dunn et al. (2023) recorded the averaged photopic OPs of the ffERG (monkey) in response to brief flashes (4.5 msec) or longer (200 msec) duration stimuli using an interstimulus interval of 2 s. In response to brief flashes, the OPs were extracted using a Blackman filter with ‐3 dB points at 93 and 212 Hz and a centre frequency of 152.5 Hz. For longer flashes, a bandpass of 0.3–300 Hz was applied. A fixed criterion time was used to measure the OP amplitudes as root mean square (RMS). The OPs were found to be associated with the b‐wave (ON OPs) at 20–60 msec (red/blue set‐up) or 15–55 msec (white/white set‐up) and with the d‐wave (OFF OPs) at 230–270 msec and 215–255 msec, respectively.

Forte et al. (2008) analysed the dynamics of the oscillatory response of the dark‐adapted ffERG (of the rat) mathematically using a continuous complex Morlet wavelet transform. Short flashes and an interstimulus interval between 5 and 90 s were used. Depending on increasing flash exposure, the OPs were found to peak earlier, and cluster at 50 msec in the 70 and 130 Hz band for moderate intensities and cluster into two groups at 20 and 50 msec in 70 Hz and 120 Hz band for higher intensities, respectively.

Very recently, an automated denoised bandpass‐filtered recording technique and analysis of the OPs of the ffERG have been reported in an animal study of normal and diabetic rats (Feola et al., 2023). However, the observed differences between automated and manual markings of the amplitudes of the OPs remain challenging and may be less meaningful to use even though the implicit time of the second wavelet (OP2) may be useful.

Most recently, Gauthier et al. (2025) used a more advanced and semi‐automatic method (Hilbert transform) to analyse the scotopic OPs of the ffERG in an experimental study (mice). Novel features of the oscillatory response to dimmer flashes splitting into two distinct bursts, different from the single burst observed for the stronger flashes, were observed. Further work is probably needed to apply this method for human patients and clinical studies, now used experimentally in rod‐dominated mice.

#### 2.1.2 mfERG

Hood (2000) recorded the averaged photopic OPs of the *mfERG* using a so‐called 7F (slow) sequence to analyse the response to hexagons presented in a pseudorandom sequence (m‐sequence) of black and white presentations. Both first‐ and second‐order kernel responses were measured. Using slow down conditions, the first‐order response may be regarded as an ‘impulse response’. Influenced by adaptation to successive flashes immediately preceding the second‐order kernel response presents additional characteristics of the mfOPs. The OP response from the parafoveal regions contained more activity. Accordingly, the OPs of the temporal retina also appeared more prominent than those of the nasal retina (Wu & Sutter, 1995), contrary to the a‐ and b‐waves, showing variation in location. The OP responses also reflected immediate flash history and adaptive mechanisms.

Fonseca et al. (2022) recorded the late appearing OPs of the photopic mfERG by using a 43° nasal/temporal array of 103 hexagons centred on a one degree fixation cross, m‐sequence and first‐order responses, to measure the implicit times of N1, P1 and N2. Retinal volume was measured by OCT and a correlation between retinal layer volume and voltage as function of time could be mathematically estimated, however, not necessarily correlated to origin in that layer.

#### 2.1.3 fmERG

Miyake (1990) recorded the photopic OPs of the averaged human fmERG with a short time constant (3 msec) and a band pass filter of 53–300 Hz using a 16‐min fixation target and analysing the spatial distribution of the OPs. The OP response being sparse in the fovea was found to be much larger in the perifovea and larger in the upper and temporal macula, respectively (Miyake et al., 1989). This was not the case in the a‐ and b‐waves. A selective reduction of the OP response of the fmERG in different clinical diseases in the macula was also described and should be useful in future studies.

#### 2.1.4 Postprocessing for OP amplitude analysis

The amplitude, power or energy of OPs is commonly used as an indicator of the overall signal strength contained in the high‐frequency OP components of the ERG. For human data, it can be estimated by bandpass filtering the ERG (typically 75–300 Hz) (Lundstrom et al., 2007) and usually by integrating the squared fast Fourier transform coefficients over the dominant frequency range (Wachtmeister, 1973b). This method has been applied both with (Wang et al., 2020) and without (Algvere & Westbeck, 1972) taking the square root. Alternatively, the summed or mean amplitudes of individual OP peaks in the time series can serve as an approximation (Lundstrom et al., 2007), potentially together with the relative amplitude of each OP (Lachapelle, 1994).

### 2.2 Optimal recording conditions

#### 2.2.1 Conditioning flash

It is well documented that the oscillatory response is very dependent on and very sensitive to changes of state of adaptation (see also overview Wachtmeister, 1998). Early work in humans reported that, in complete dark adaptation, no clear and distinguishable oscillatory peaks were observed in response to the first single flash stimulation in DA (Algvere & Wachtmeister, 1972; Algvere & Westbeck, 1972). Therefore, a so‐called ‘conditioning flash’ technique may be used to optimize the oscillatory response of the ffERG when recorded in dark adaptation. The ‘conditioning flash’ will induce a mesopic adaptational state at which it is known that the OPs are most easily elicited reflecting an interaction between the rods and cones (Wachtmeister, 1972). To maintain a steady state of adaptation during the time of recording, usually successive flashes are given with constant interstimulus interval and constant stimulus intensity. The conditioning first flash will diminish the rod contribution to the response to the following flash in the dark.

Later studies showed that there are indeed two low amplitude oscillatory peaks present in the single response to the first flash recorded in complete DA (Lundstrom et al., 2007). Furthermore, in mesopic background light, the morphology altered in the first single response to the first flash.

If not a steady state of adaptation is kept, the OPs may dramatically change in shape, implicit times, dominant frequency and energy content (Wachtmeister, 1973b). For instance, the dominant frequency of the OPs decreases from about 150 Hz in a relatively more dark‐adapted (scotopic) condition to about 105 Hz in a relatively more light‐adapted (photopic) state using, that is, single flash stimulation and shorter interstimulus interval in the dark (Wachtmeister, 1972). See also previous section.

#### 2.2.2 Mesopic adaptational conditions

From earlier, both clinical and experimental work (el Azazi et al., 2004; Wachtmeister, 1972, 1973a, 1974a, 1974b, 1974c, 1986; Wang et al., 2001; Wang, el Azazi, et al., 2015), as mentioned, we know that the OPs reflect both scotopic and photopic activities of the retina and are related not only to cone function but also to rod function. They are most easily elicited when a reorganization of the retinal pathway corresponding to the threshold of the cones has occurred and the rods are more sensitive than the cones, that is, at a mesopic state of adaptation. Furthermore, the oscillatory response can be optimized by exposure to a steady background illumination of mesopic intensity that corresponds to approximately moon light illumination (10^−2^–10^0^ cd/m^2^). Moreover, an oscillatory response of maximum amplitude can also be obtained during adaptation to darkness reaching the mesopic level (the transition from the cone to the rod vision, the Kohlrausch kink) after pre‐exposure to a bright background light. Those two last options may though be difficult to apply in a clinical non‐experimental set‐up.

Therefore, in clinical practice, it has been and is advisable to, in a simple way, obtain optimal OPs to record single responses in dark adaptation using a short bright stimulus flash of rapid rate of rise, a conditioning flash inducing a mesopic state of adaptation and keep a constant interstimulus interval, for example, 30 s. In this context, it may also be added that, in some clinical situations and studies, it will be beneficial to record low‐amplitude scotopic OPs and thus use very dim flashes and longer interstimulus intervals; see also above (Kim et al., 2018; Pardue et al., 2014).

### 2.3 Non‐bleaching neuronal (network) adaptation

Neuronal (network) adaptation represents a swiftly acting system relying on activity in a retinal network comprised of a series of neuronal events occurring in the retina (see also Section 1.1). The OPs have been shown to reflect radial flows of currents, probably in inhibitory feedback neuronal pathways, in the inner retina (see also Section 3). An important observation is that, for optimal recording conditions of the OPs, short high‐intensity flashes of rapid rate of rise that bleaches negligible amount of pigment are used (Ripps & Weale, 1969; Rushton & Baker, 1963; Wachtmeister, 1972). Therefore, the recording of the OPs may be a convenient mean to study and analyse neuronal adaptive capacity of the inner retina that is not regulated by bleaching of photopigments and thus does not reflect a photochemical process of the photoreceptors.

Neuronal adaptation occurs within a very short time frame, typically within hundreds of milliseconds after a conditioning flash or change in background illumination contrasting with the slower photochemical adaptation that requires minutes (Lundstrom et al., 2007; Wachtmeister, 1973b). In such a set‐up, the slow potentials (a‐ and b‐waves) are very little influenced by the previous (conditional) flash used but greatly affect the energy and dominant frequency of the OPs. OP energy (see also Section 2.1.4), using Fourier approach (Algvere & Westbeck, 1972), can increase 10‐fold while a‐ and b‐waves remain unchanged or decrease slightly (Wachtmeister, 1973b, 1998). The dominant frequency of the OPs then shifts from about 150 Hz in scotopic conditions towards about 105 Hz in photopic states (Wachtmeister, 1973b, 1998). Thus, OP analysis can serve as a sensitive marker of neuronal adaptive capacity of the retina. Key indicators include amplitude or energy changes (OP1‐OP4), latency shifts and frequency domain alterations under controlled adaptational states. Failure of OP amplitudes or energy to increase under mesopic conditions, or dominant frequency to shift changing from scotopic to photopic conditions or vice versa, indicate impaired neuronal adaptive function.

Moreover, the spectral sensitivity of the OPs has been found to have two peaks (472 nm and 551 nm, respectively) and lack a Purkinje shift (Wachtmeister, 1974a, 1974b). The slow potentials (the a‐ and b‐waves), on the contrary, change from a predominantly photopic shape of spectral sensitivity to a scotopic one in the same study. A further indication and evidence for the absence of a direct involvement of photoreceptor activity, pigment bleaching and photochemical adaptation to be expressed in the OPs and the OPs to reflect neuronal adaptive activity in the retina.

The spatial summation of the OPs represents a broader area of activity than that of the a‐ and b‐waves also wider than that of the extracellular response of the amacrine cells in the amphibian retina (Hahn & Wachtmeister, 1989; Wachtmeister, 1986; Wachtmeister & Hahn, 1987). The range of (spatial) summation also varies with adaptational conditions, being reduced in light adaptation, probably activated by the amacrines initiating a series of neuronal events—reflecting a non‐bleaching adaptational function of the retina.

Freitag et al. (2024) studying the ramp‐after effect in 20 healthy adults using a sawtooth‐modulated light for upwards and down ramp adaptation recorded long flash ffERG to probe the neuronal adaptive state. They found significant and non‐significant changes in the OP response (OP1, OP2 and OP3), but no opposite sign‐reversing connections. Downwards adaptation being stronger but different between the individual OPs. Thus, additional evidence and further indication of neuronal adaptive relay stations and modulation of intraretinal processing seem to occur in the inner retina and expressed in the oscillatory response.

To conclude this section (2nd), there are several methods and techniques in practice to record, describe and analyse the OPs. Clinical and experimental studies have been focusing on different retinal areas and parts of the retina, full field, posterior pole, macula, different layers, etc. It may (therefore) be difficult to in an easy way compare and evaluate findings, results and conclusions. However, there are several grounds to deduce that the oscillatory response may be used to evaluate and early detect disturbances in the neuronal adaptive capacity of the retina.

## 3 ORIGIN OF THE OPS

Different approaches to investigate the origin(s) of the OPs of the retina have been used. Primarily microelectrode studies and pharmacological analyses have been performed to explore the underlying cellular mechanisms of the OPs. Genetic models have also been developed and used in order to experimentally elucidate their origin(s).

### 3.1 Microelectrode studies

Early work by Ogden (1973) studying the laminar profile of voltage of the retina of the monkey implicated the level of origin of the OPs in the inner plexiform layer (IPL). Later, microelectrode studies (Wachtmeister & Dowling, 1978) of the cold‐blooded animal (mudpuppy) showed that the OPs reflect radial flows of current(s) within the retina and representing a series of radial loops moving from the inner to the outer retina to be explained by a feedback system and also suggesting separate origins of the individual oscillatory peaks. Heynen et al. (Heynen et al., 1985; Heynen & van Norren, 1985a, 1985b) performed a current source density study in vivo in the monkey retina and found that, in the primate retina, the origin of the OPs reflects activity of the bipolar and amacrine cells.

Electrophysiological studies of the human retina (Tremblay & Lam, 1993) later corroborated the view (Wachtmeister & Dowling, 1978) that there are different generators of the individual OPs of the ffERG also in man. Moreover, clinical studies of retinopathies indicated different cellular origins of the peaks; the earlier OPs to be related to the rod system and the later ones to the cone system, respectively (Heckenlively et al., 1983; Lachapelle et al., 1983).

Furthermore, the early OPs of the photopic mfERG and ffERG seem to be associated with the ON pathway, and moreover, a clear discrepancy of the ON and OFF mfOPs comparing the centre and peripheral ring responses in complete congenital stationary night blindness (cCSNB) was reported (Dorfman et al., 2020).

### 3.2 Pharmacological studies

Many experimental studies of the drug effects of the conventional ffERG and mfERG have been executed to better understand the cellular contribution to the neuronal component of the ERG, including the OPs (Dong et al., 2004; Harada et al., 2013; Hare & Ton, 2002; Hood et al., 2002; Liu, Pettersen, et al., 2014; Liu, Zhou, et al., 2014; Rangaswamy et al., 2003; Sakai et al., 2009; Ueno et al., 2004; Wachtmeister & Dowling, 1978).

#### 3.2.1 Various pharmacological neuroactive agents

Dong et al. (2004) used band filtering 100–1000 Hz, measuring the OP power response area by converting all the negative voltages to positive ones, integrated the rectified response over fixed time periods of averaged bright flash photopic ffERG in rabbits. The mechanism of the OP generators was analysed by quantifying the effects of pharmacological neuroactive agents (APB, TTX, PDA, CNQX) blocking synaptic transmitters and channels (voltage gated). They described the early OPs likely to be generated mainly by photoreceptors, the intermediate ones by action potential independent and the late subgroup by action potential dependent mechanisms in the ON pathway in the inner retina in the rabbit.

Rangaswamy et al. (2003) studied the slow sequence mfERG, closely resembling the photopic ffERG, using pharmacological agents intravitreally to separate the contribution of the different retinal cells in the monkey. They confirmed the nasotemporal asymmetry of the oscillatory response and described the spike‐driven oscillatory activity to be prominent in the centre and temporal retina. Furthermore, the OPs were eliminated by blocking the ON pathways and concluded that the photopic ffERG was mainly shaped by the stimulation of the more peripheral regions of the mfERG response.

#### 3.2.2 Glutamate/glycine

Ueno et al. (2004) used a glutamate‐agonist (APB) and a glutamate‐antagonist (PDA) analysing the mechanism of the photopic hill of the b‐wave in response to the photopic flash in the adult monkey ffERG. APB, blocking the ON neuronal pathways (bipolar cells, as well as third‐order neurons, amacrine and ganglion cells) completely blocked all the oscillatory peaks. PDA, an OFF bipolar cell blocker (and also an ON pathway blocker of the third‐order cells—likely being an OFF pathway effect) attenuated the early oscillatory peaks and completely blocked the later peaks of the OP response. They (Ueno et al., 2004) also mathematically extracted and analysed the so‐called ‘subtracted ON component’ (APB‐sensitive) as well as the ‘subtracted OFF component’ (PDA‐sensitive) and showed that the OPs present and OPs gone, respectively.

Another glutamate‐agonist (NMDA) removing inner retinal neuronal influences (amacrine and ganglion cells) has been described to ‘remove the OPs of the leading edge of flash response in the primate (monkey) mfERG’ (Hood et al., 2002). Another indication of at least the early OPs seem to reflect activity of the ON‐pathway.

A selective inhibitor of a glycine transporter (GlyT1) increasing glycine concentration has been associated with visual disturbances in humans and alterations in the OP response of the scotopic ERG in the rat (Liu, Pettersen, et al., 2014). Glycine acting as a co‐agonist at NMDA receptor modulating glycinergic neurotransmission (probably in the glycinergic amacrine cells in the inner retina) and indirect glutaminergic neurotransmission (at bipolar and ganglion cell level) or together were shown to play a critical role in the generation of neuronal activity in the inhibitory retinal circuits expressed in the oscillatory response. Therefore, the OPs seem to be functionally linked with the amacrine cells expressing the glycine transporter (GlyTI).

To summarize shortly, the later oscillatory peaks relate to, are present and seem to depend on a functioning neuronal OFF pathway in the inner retina. Both intact ON and OFF pathways seem to be a prerequisite for the generation of the oscillatory response. Furthermore, this is in accordance and corroborates the view first suggested by Wachtmeister and Dowling (1978) that the early and later peaks having different neuronal origins.

#### 3.2.3 GABA/dopamine

Several pharmacological studies have reported that the OPs are selectively depressed by inhibitory synaptic transmitters (i.e. GABA and dopamine) in the inner plexiform layer blocking particular cells and circuits in the retina (Dai et al., 2017; Hood et al., 2002; Kim et al., 2018; Wachtmeister & Dowling, 1978; Wang, Mojumder, et al., 2015).

Early pharmacological investigation of the mudpuppy retina (Wachtmeister & Dowling, 1978) showed that the OPs of the transretinal ERG were selectively depressed by inhibitory synaptic transmitters like GABA and dopamine. Moreover, a post‐intravitreal injection of GABA was reported to cause a loss of the OPs in mice (Saszik et al., 2002). Furthermore, a GABA‐sensitive oscillatory component of the second‐order kernel of the mfERG in the monkey has been reported to be removed by dopamine, at least up to the peak of P1 (Hood et al., 2002).

Wang, Mojumder, et al. (2015) not only focused on different GABA receptors (GABAR), mediating feedforward and feedback inhibition of signals in the amacrine cells, but also expressed in the horizontal and ganglion cells, in the rodent (mice) retina. The GABAaR is known to mediate a fast transient, quickly desensitizing response and GABAcR to mediate a slow sustained, slowly desensitizing response as well as a tonic current. They described that baclofen (a GABAbR‐agonist) significantly enhanced the OPs of the scotopic and photopic ffERG. Moreover, a GABAcR antagonist (pharmacological blockade of GABAcR) or genetically deleted GABAcR caused the OPs to increase, the b‐wave to decrease and left the a‐wave unaltered. After pharmacological blockade of GABAaR, b‐wave peak narrower and slow oscillations occurred in the DA ERG. Thus, the oscillatory response seems to be strongly modulated, relies on and expresses activity in the inhibitory part of the microcircuitry in the inner retina—also independently of the b‐wave (bipolars) and the a‐wave (photoreceptor) generators.

Dai et al. (2017) studied the effect of different neurotransmitters and their antagonists on the dark‐adapted ffERG in the rat using the GABOR function for analysis (amplitude and time parameters) and performing a Fourier transform frequency domain analysis. The later OPs were described to be removed (the b‐wave abolished and the OP/b‐wave ratio to increase) by GABA. The earlier OPs did not appear to be significantly altered. Furthermore, the later OPs were increased by GABA combined with TPMP (a GABA antagonist), that is, the OPs were restored by the effect caused by GABA. Dai et al. (2017) also suggested that the later wavelets to be generated by the activity in the ON pathways. Moreover, they concluded that the GABAa and GABAc receptors as well as glycine receptors seem to regulate the low‐frequency (later) component (s) of the OP response and the GABAa receptors to modulate both the low (75–80 Hz) and also the high (130–150 Hz) frequency components of the OP response. Thus, also indications in this context that GABAa receptors may respond more quickly to GABA than the GABAc receptors seem to do. Furthermore, strychnine (a glycine antagonist) was described by them to reduce the OPs and reduce the OP/b‐wave ratio and mainly affecting the low‐frequency component of the OP response of the rat ERG (Dai et al., 2017). They concluded that these results indicate that AII amacrine cells may contribute to the OPs by modulating the input to rod bipolar cells via GABA/Glycine receptors by inhibition of both glycine and GABAa receptors decreasing the electric response of the rod bipolar cells.

Kim et al. (2018) quite recently reported that, in genetically modulated mice (tyrosine hydroxylated enzyme deficit) with a chronic reduction (80%) of retinal dopamine, a delay of the OPs in the ffERG in response to *dim* stimulus flashes was observed.

Thus, dopamine seems to play a most sensitive and integral role in the neuronal cell circuits, especially in the scotopic rod‐driven ones, expressed in the oscillatory response. Tentatively through a co‐release of dopamine by GABAergic inhibitory amacrine cells.

In summary, several neurotransmitters and neuronal pathways seem to be involved in the generation of the OPs, ON and OFF pathways, primarily inhibitory ones and most probably initiated by the amacrines in the inner plexiform layers. Thus, the OPs seem to play an important role in visual signal processing.

### 3.3 Genetic animal models

Lei et al. (2006) turned to genetically modulated mice to assign a class of photoreceptors to generate one or more of the OPs. They used two mice models possessing pure rod function (cone receptor function loss, *cpfl 1* mouse) and pure cone function (rhodopsin knock‐out *rho−/−* mouse), respectively. Fast Fourier transforms (FFT) were performed to determine the total power of the OP signal, its frequency spectra and time latencies of the ffERG. The short latency OPs were reported to be rod driven and the long latency OPs to contain both rod‐ and cone‐driven signals.

The transgenic (tg) rabbit model (tg rhodopsin P 347 L) with a reduced rod photoreceptor function and degeneration was used by Sakai et al. (2009). In young tg rabbits, the oscillatory response of the photopic ffERG was supernormal, the a‐wave was significantly smaller and the b‐wave was not changed compared with WT (wild type) ones. The later three OPs (OP2–OP4) were significantly larger, but OP1 was not different between the two types of rabbits. In the tg rabbits, TTX (a glycine antagonist, blocking the action potentials produced by ganglion cells and certain classes of amacrine cells) was shown to cause a more pronounced reduction of the oscillatory response (65%), especially the later (OP3–OP4), in comparison with the WT ones (28%). The scotopic OPs were not significantly changed and neither was the first OP (OP1). They concluded that the photopic OPs originate mainly from the third‐order neurons of the inner retina, predominantly the ON pathway and not with a large contribution from the ON/OFF bipolar cells. The supernormal OPs observed in the tg rabbits seem to reflect activity in TTX‐sensitive neurons, either amacrines or ganglion cells.

In addition, at an early stage of disease in dystrophic RCS rats, Harada et al. (2013) reported that NMDA (an excitatory amino acid agent suppressing the synaptic transmission between the bipolar cells and the third order neurons) caused a decrease of the oscillatory response. They corroborate the view that NMDA has a role in the generation of the OPs and concluded that the OPs seem to serve as a sensitive indicator of photoreceptor degeneration.

Concisely, studies of genetically modified rodents, some also combined with pharmacological experiments, indicate that the short latency OPs are rod driven and the later ones are cone driven and that the photopic OPs seem to originate predominantly from the ON pathway, without a large contribution from the ON–OFF neuronal pathway.

To conclude this section (3rd), much work has been devoted to try to pinpoint the precise and more exact cellular origin of the small wavelets, the OPs. Several types of receptors, neurotransmitters, neurons, neuronal pathways, etc., in the microcircuitry of the inner retina seem to be involved in this process building up the neuronal, non‐photochemical adaptive function of the retina expressed in the OPs. However, it remains still a challenge to identify the exact specific neuron(s) for their origin(s). Much evidence, early and more recent one, have been presented and may be summarized below:

1. The OPs seem to have multiple generators and the individual oscillatory peaks have different and separate origins. The very first wavelet may be coupled to the a‐wave and be photoreceptor‐driven, the earlier rod‐driven and the later cone‐driven.
2. Some peaks, most possibly the later ones, are associated with OFF bipolar cell activity and may vary between different species. Photopic OPs have been described to originate predominantly from the ON pathway. Both ON and OFF bipolar cell neuronal pathways seem to be a prerequisite for an oscillatory response to be generated. However, the ON–OFF bipolar cells seem **not** to make a large contribution to the generation of the OPs.
3. The oscillatory response represents activity in inhibitory, probably dopaminergic‐GABAergic, neuronal feedback pathways initiated by glycinergic type of amacrine cells, tentatively AII type, in the inner plexiform layer.
4. Different GABA receptors are or seem to be involved, that is, most probably the GABAaR receptor, decreasing the electrical response to rod bipolar cells.
5. The OPs seem to play an important role in visual processing and neuronal adaptive activity. Its most vital function, in most and may be all, animals including man, seems to be for protection and survival when a need to quickly adapt and adjust from dark to light and vice versa.

## 4 DEVELOPMENT OF THE OPS

This part outlines the normal course of development of the oscillatory response across the human lifespan reflecting the maturation and final refinement and ageing of the inner retinal microcircuitry. Thus, it presents development from preterm, through postnatal, to advanced age.

### 4.1 Preterm/Premature

Berezovsky et al. (2003) studied the maturation of the OPs of the standard averaged DA ffERG in very low‐birth‐weight infants with no signs of ROP (Retinopathy of Prematurity) relating the function with the changes in morphology (cone density, elongation of photoreceptor outer segment). Healthy preterm infants (*n* = 17) were examined their first months of life, at 3 and 8 weeks (mean adjusted ages). Very small maturational changes—and not in the OPs—were found comparing the two age groups. There were only an increased amplitude of the single‐flash cone‐b‐wave response and 30 Hz flicker, respectively. They concluded that OPs were found to mature at an independent rate and faster compared to the a‐ and b‐waves. They also implied that there is an earlier maturation of the inner retina (Mueller cells/bipolars) compared to the outer retina (photoreceptors) and that the rod‐mediated function is maturing earlier than cone‐mediated function.

Later, Mactier et al. (2013) studied dark‐adapted OPs of the ffERG in preterm infants (*n* = 38) less than 31 weeks gestational age and/or birth weight of 1250 g or less and also at out‐patient follow‐up at 10 weeks of age. In the group of infants without ROP (20 of 38), the earliest appearance of an oscillatory response was observed at 30 weeks +5 (postmenstrual age, PMA) and in one infant at 32 weeks +2 (PMA). At 7.5 weeks postnatally, there was a 50% likelihood of OPs present, and OP2 grew rapidly thereafter and more than OP3 and OP4. All infants at 40 weeks (PMA) showed OPs. Amplitudes grew and peak times of the individual OPs decreased with increasing maturity. OP1 seems to be less likely present in infants with developing ROP. There were smaller OPs at 37 weeks PMA and significantly smaller at 50 weeks PMA for threshold ROP.

In summary, there is indicative evidence that, in humans, the rate of preterm and premature development of the OPs seems to occur independently compared to the functions of the outer retina and that the rod‐mediated OPs to mature earlier than the cone‐mediated ones. The earliest appearance of an oscillatory response was observed at 30 weeks of age. The growth of OP2 was larger than that of OP3 and OP4.

### 4.2 Term born/ postnatal/ infancy

In 2005, Moskowitz et al. (2005) stated that the OP amplitudes of the ffERG are a late maturity feature of retinal function in their study of term born 10‐week‐old infants (*n* = 15). Infant OPs were significantly smaller, less mature than adult ones. The immaturity of the scotopic responses was more marked (19% of adults) than the photopic ones (47% of adults). The earlier wavelets were more immature than the later ones. There was no significant variation in number nor in implicit time in the scotopic OPs. However, the implicit time of the photopic peaks was significantly longer. Furthermore, the interpeak time and the periodicity of the OPs were not different between the infants and the adults (frequency of 155 Hz in scotopic and 135 Hz in photopic conditions). The OPs were relatively more immature than the photoreceptor response (a‐wave). They concluded that there was no evidence of the immaturity of the OPs to be related in a simple way to the immaturity of the photoreceptors.

Term born infants (*n* = 38) in good general health (age 63–78 days) were examined using a discrete Fourier transform (DFT) method and described in a study of the development of the dark‐adapted OP of the ffERG in ROP by Akula et al. (2007). OP power spectrum, energy, dominant frequency and sensitivity were evaluated. The amplitudes of the OPs were small (median 5 μV), similar to a root mean squared noise (about 3 μV). There was a single peak in the frequency domain and a dominant frequency as expected from the interpeak interval (127 Hz) and thus different from the adults having a frequency domain that was bimodal with peaks at 100 and 140 Hz. The OP energy of the infants was also lower than the adults.

Several animal studies have been published regarding the postnatal development of the oscillatory response. In the context of maturation and behaviour of the OPs in background adaptation and readaptation to the dark, there is experimental evidence that the early postnatal development of the fine‐tuned interaction between the rod and the cone system and the neuronal adaptative function of the retina as expressed in the oscillatory response lags behind that of the photoreceptor response and is delayed to that of the bipolar function (Wang, El Azazi, et al., 2015). The process of adaptation to a mesopic background light and recovery in the dark after exposure to a mesopic background light in baby rats was found to be different to the mature rats and to the adaptive behaviour of the a‐ and b‐waves. Thus, this important aspect of activity of neuronal adaptive function was found to be more immature than that of the photoreceptor one as reflected in the a‐wave.

It has also been reported that, in mice, light deprivation from birth temporarily delays the postnatal developmental increase of the a‐ and b‐waves, but persistently suppresses the OP amplitudes (Tian, 2004; Vistamehr & Tian, 2004). Findings indicate that there is a functional activity‐depending plasticity also in the inner retina as expressed in the oscillatory response. Furthermore, the reduction of the OP response recovered to control level after return to cyclic light, but the younger animals required a longer time, that is, weeks.

Joly et al. (2006) studied the structure and form of the consequences of postnatal exposure to bright light in rats (1 month and 2 months old). They described a tendency of the amplitudes of the photopic and scotopic OPs to be larger in juvenile rats than those of the adult controls. The oscillatory response was also found to be better preserved in the younger than in the adult animals. The rod‐mediated OPs were more affected than the cone‐mediated ones.

Briefly, at a later stage of development, at post‐term, the earlier wavelets and the scotopic OPs were less mature than the later and photopic ones, respectively, and also more immature than the photoreceptor response. In infancy, the dominant frequency of the OPs was different from the adult, containing one single peak in the frequency domain and thus contrary to the adults showing two peaks. There is also experimental evidence (rats, mice) that the maturation of the background light adaptive function expressed in the oscillatory response lags behind that of the photoreceptor one and there is a functional depending plasticity during development.

### 4.3 Adult/ageing/old age

The changes with age of the multifocal OPs (first‐ and second‐order kernel) were studied in human subjects (*n* = 58), 13.6–58.8 years old, by Kurtenbach and Weiss (Kurtenbach & Weiss, 2002). The amplitudes significantly decreased, and the implicit times increased with age. The third OP (second‐order kernel) was the largest one and showed the most pronounced changes with age. Latencies of the peaks were delayed and were most affected by age. The rate of change of the amplitudes of the first‐order kernel was largest. The later the peak, the higher its rate of change. Changes across the retina (1.8–30°) were also observed. The amplitudes appeared smaller, and the implicit times decreased in the periphery. One exception was peak 2 of the first‐order kernel, which did not show a significant change with rings.

Dimopoulos et al. (2014) studied the influence of age of both the dark‐adapted (DA) and light‐adapted (LA) ffERG in healthy subjects 20–82 years of age. The OPs were measured in time/amplitude domain as well as analysed using a Morlet wavelet transform (MTW) method to document the time–amplitude–frequency distribution. The age‐related changes of the oscillatory response preceded the a‐ and b‐waves (the photoreceptor and postreceptor/bipolar ones). By 40 years of age, there was a reduction in both the DA‐ and LA OP amplitudes and the implicit times were prolonged. There was a linear association between the attenuation of DA OPs amplitudes and age. High‐frequency (150–155 Hz) OPs were unaffected by age. There was a reduction of power at 60 years of age. MTW revealed a high frequency band (135 + − 6 Hz) locked to the early OPs and a low frequency (82 + −7 Hz) related to the onset of the early and late OPs.

Jung et al. (2024) using a continuous wavelet transformation method (Righetti et al., 2021) evaluated the age dependency of the ffERG in healthy (*n* = 73, 14–73 years old) individuals. The power of the OPs was analysed and displayed a high variability with a small reduction with age not statistically correlated, but with a corresponding significant delay.

In brevity, the function of neuronal adaptation as expressed in the OPs undergoes age changes relatively early in life, already at about 40 years of age and seems to precede rod and cone dysfunction. The OPs are therefore highly sensitive and seem to detect early ageing.

Moreover, very recently Penaloza et al. (2025) reported a significant age‐related decline psychophysically, testing vision (reading) under mesopic and photopic conditions in normally sighted individuals (*n* = 157). They interestingly observed a more marked decline under mesopic light. Tentatively such more pronounced mesopic age‐related decline may well be expressed in the oscillatory response that sensitively reflects the neuronal adaptive activity and the interaction between the rods and cones, that is, in mesopic conditions.

To conclude this section (4th), focusing on the development and ageing of the OP response:

1. There is an independent development, maturation and ageing of the oscillatory response unrelated to the photoreceptor and bipolar ones as measured by the a‐ and b‐waves.
2. Preterm and post‐term development of the individual OPs seems to differ in rate and priority of maturation.
3. The neuronal adaptive system as reflected in the OPs sensitively detects ageing.
4. Experimental data indicate that there seems to be postnatal plasticity in the inner retina as expressed in the oscillatory response and thus the neuronal adaptive function.

## 5 DISEASES AND DISORDERS

The oscillatory response is altered in a wide range of retinal diseases, reflecting dysfunction in both neuronal and vascular components of the inner retina. This section reviews how the OPs are affected in inherited and acquired conditions, including retinal dystrophies, vascular retinopathies and mixed or toxic disorders. By examining disease‐specific patterns of OP changes, insights can be gained into underlying pathophysiology and potential diagnostic or prognostic value.

### 5.1 Neuronal diseases

It is well known that, for example, most hereditary retinal disorders primarily affect the photoreceptors and pigment epithelium. However, there are also indications that neuronal pathways in the inner retina, like alterations of On–Off response amplitudes and implicit times, and structures like bipolar, amacrine, horizontal and ganglion cells are involved (Ruether & Kellner, 1998). A mild and moderate loss of inner retina usually seems to occur early in time, like in stationary retinal degeneration and later in progressive types of retinal dystrophic retinopathies. This survey will only focus on neuronal diseases with dysfunction of the inner retina indicated by altered oscillatory responses.

#### 5.1.1 Inherited retinal degenerations (stationary or progressive)/retinal dystrophic retinopathies

Already in the 1990s, detailed descriptions of different hereditary retinal degenerations and dystrophic retinopathies with loss of early and late OPs (Heckenlively et al., 1983) and relative behaviour of the amplitudes of each OP in suprathreshold photopic ERG (Lachapelle, 1994) have been published as well as presented in a previous survey (Wachtmeister, 1998).

In this overview, the retinal dystrophies will be presented based on rod, cone or mixed rod/cone way dysfunction.

First, one example of inherited retinal degeneration with dysfunction of the rod pathway in congenital stationary night blindness (CSNB) of the Schubert–Bornstein type will be described. Second, hereditary retinal dystrophies with alteration of the function of the cone pathway exemplified by achromatopsia and monochromatopsia will be characterized. Finally, hereditary retinopathies involving the inner retina and the inner plexiform layer, processing signals in both the rod and cone pathways, like in retinitis pigmentosa (RP) (rod/cone, cone/rod), and congenital retinoschisis will be shown.

##### Rod pathway dysfunction

In 2008, Audo et al. (2008) presented a comprehensive review of inner retinal dysfunction including examples of photopic ffERG in congenital stationary night blindness (CSNB) of the Schubert–Bornschein type (post photo‐transductional type)—a classification introduced by Miyake et al. (1986).

Audo et al. (2008) presented ffERG in response to photopic single flashes in a patient with x‐linked complete CSNB (XLCSNB) with a selective loss of both rod and cone ON‐function but both OFF pathways preserved. The first two OPs (OP1 and OP2) on the ascending b‐wave were reported to be absent and further closely looking at the recording (figure 2A) late wavelets evoked after the b‐wave described by Lachapelle (1990) seem to remain. In a milder degree of CSNB, x‐linked incomplete (XL‐CSNB2) with both ON and OFF response abnormality, both the early and late OPs seem to be absent (Audo et al., 2008 figure 2B). In the rarely occurring autosomal recessive CSNB with selective loss of the ON response and variable cone function the late OPs, like in CSBN1, seem in this case to remain (Audo et al., 2008 figure 2C).

More recently, in 2020, Dorfman et al. (2020) used mfERG to analyse the centre‐periphery distribution of the mfOPs in patients with complete (c) CSNB (*n* = 5). They described that the responses originating from the most central ring disclosed ‘a near normal presence of the early mfOPs (OP2, OP3, OP4) and remaining later OPs’. There was a discrepancy compared with the responses from the more peripheral rings, being devoted of the early OPs (OP2 and OP3) (Dorfman et al., 2020 figure 2). They concluded and suggested that the fovea possibly may function normally at least at the ON and OFF‐pathway(s) level indicated by the near normal mfOP response in the centre ring—even though there was a malfunction of the ON pathway in cCSNB.

In Best's disease (autosomal dominant), a pathological oscillatory response affecting the earlier OPs of the ffERG, similar to the one in CSNB, has been observed (Lachapelle, 1994).

Another inherited disease (autosomal dominant), snowflake vitreo‐retinal degeneration (MIM 193231), with variable ffERG the photopic OPs have been reported to be undetectable (Hirose et al., 1980).

Shortly, in retinal dystrophies and degenerations with rod pathway dysfunction and abnormal ON and OFF responses (XL CSNB 2), the photopic OPs seem to be absent, but in case of abnormal scotopic system with preserved OFF pathway function (XL CSNB 1 and autosomal recessive CSNB), there seem to be remaining late OPs. Different synaptic mechanisms transmitting signals to bipolars and further in the inner retina seem to be used by the cones in the centre compared to the periphery of the macular area as reflected in the oscillatory response. Analysis of the oscillatory response may thus be of further clinical relevance in differentiating between variable phenotypes of hereditary rod pathway dysfunction.

##### Cone pathway

Jiang et al. (2024) presented recordings of one patient with genetically confirmed (autosomal recessive) achromatopsia (ACHM) in their study of the cone‐driven strong flash ffERG and negative ERG in a large (more than 200) cohort of healthy adult volunteers (TWIN UK). They used a blue background light to selectively lowering rod function and induce a response closer to reflecting the normal dark‐adapted cone system response including the oscillatory peaks. Strong white stimulus flashes were used and in dark adaptation elicited an oscillatory response with six wavelets which were substantially reduced in number and amplitude in white background light. In the rod‐saturating blue background light, all OPs were further diminished or non‐recordable. However, one may argue that, in figure 3 (Jiang et al., 2024) in the recordings of the young patient with ACHM there seems to be very low subtle amplitude wavelets remaining in dark adaptation as well as in the rod‐saturating background. As the authors suggested, one reason may be that some degree of adaptation of the cone system and/or residual rod responses may still be present. In brief, there seems to be recordable but very low amplitude OPs in the ffERG in ACHM.

Righetti et al. (2021) studied the OPs of the ffERG in 41 achromatic persons (autosomal recessive) using Morlet complex wavelet transforms in order to investigate the connection between the inner retinal network and the photoreceptors. Time–frequency maps disclosed two main bands at 70–100 Hz and above 100 Hz within 50 msec. They described a strongly reduced but not absent activity of the OPs, that is, that the time–frequency domain of the OP response, which mostly is due to the cones and a small contribution by rods, was affected. The Morlet spectrogram of the oscillatory response may therefore be used to indicate the functional status of the connections of the cones to the inner retina in ACHM. After extraction and filtering the responses, the OPs were described to be delayed and reduced in amplitudes in the 150–200 Hz frequency but did not differ in lower frequencies. In short, in ACHM, rod monochromasia, only the frequency range corresponding to the OPs was affected, but lower frequencies did not differ.

More recently, Righetti et al. (2024) presented a study of the importance of functioning cone pathway to the formation of dark‐adapted ffERG by extracting the OPs and performing a time–frequency analysis using a continuous complex Morlet wavelet convolution. Thirty‐nine genetically confirmed patients with ACHM and 20 patients with blue cone monochromacy (BMC) (x‐linked recessive) were analysed and compared to controls. Central foveal thickness and age dependence were also evaluated. Significantly reduced but nevertheless present OPs were observed. The peak power within time and frequency domains of the OPs comparing the ACHM and BCM groups did not statistically differ. The OPs were not significantly related to the a‐ and b‐waves. There was no significant relationship between the power of the OPs and the morphological structures of the retinal layers. To summarize, there are discernible OPs but of low amplitude and delayed in ACHM and BCM. S‐cones per se seem not to influence the complex OP response.

In x‐linked retinoschisis (XLRS), affecting the cone‐rich macula, the later OPs in the ffERG is known to be relatively more diminished (Lachapelle, 1994) and described to be absent in the fmERG (Miyake et al., 1993). Later, Azevedo et al. (2024) also reported abnormal OPs in the mfERG and the ffERG in a rare case of XLRS.

To conclude, the OPs may be used as a sensitive instrument for early detection of a dysfunctional cone pathway in the inner retina and a sign of mistuned and unbalanced interaction in the neuronal adaptive network in the inner retina.

Moreover, studies of gene therapy for ACHM using animal models (sheep, mice) have been performed (Banin et al., 2015; Muhlfriedel et al., 2017) and retinal function evaluated with electrophysiology. To improve and refine the objective analysis of functional outcome in these types of studies, an option may tentatively be to include the measurement of the oscillatory response.

Advanced recording techniques, including single cell ones, for mammalian retina may also develop. Such approach will hopefully enable better understanding of the converging and integrating process that occurs in the inner retina and that governs, for example, ganglion cells and further signals to the brain. Most recently, an adaptive developmental mechanism that changes the output of the ganglion cells if using full‐field flashes—but not in response to pattern (checkerboard) stimulation—has been reported in albino mice by Chotard et al. (2024). Similarly, the OPs, indicating neuronal (adaptive) activity, is known to be absent and unrecordable in the pattern ERG but clearly present in the ERG response to full‐field flashes.

It is well known that patients with achromatopsia/monochromasia often may have disturbing photophobia. A bold suggestion may be that this clinical sign may be expressed in the altered oscillatory response reflecting a lost or diminished neuronal adaptive function.

##### Mixed rod/cone pathway dysfunction

Retinitis pigmentosa (RP), a group of inherited retinal degenerations and genetically heterogeneous, involves more than 60 gene loci, is characterized by loss of photoreceptors followed by widespread and extended atrophy of the retina. It starts with progressively impaired function in the rod‐dominating periphery, subsequently and finally including the central cone‐rich retina, which usually is better preserved in the early stages.

Since the 1990s (Cideciyan & Jacobson, 1993; Hood & Birch, 1996; Lachapelle, 1994), it is well known that the OPs of the ffERG in RP are diminished in amplitudes and delayed and thus reflecting a mixed rod‐cone pathway dysfunction in RP. Loss or reduction of individual oscillatory peaks, probably a sign of abnormal wiring, for example, neurite sprouting, in the inner retina, have also been reported (Fariss et al., 2000; Li et al., 1995).

Early studies (Falsini et al., 1994) of the fmERG in RP patients also described that not only the outer retina but also the inner retina contributes to the retinal dysfunction in eyes with RP. Later, Ikenoya et al. (2007) used fmERG to investigate 39 patients with early‐stage RP and good visual acuity (1.0 or better). The OPs clearly signalled a disruption in inner retinal layers and that the oscillatory response in the macula was relatively better preserved than that of the a‐ and b‐waves. The OP changes could not simply be explained by decrease in sensitivity to light in RP patients. The selective preservation of the OPs in peri‐ and parafovea was interpreted to probably be due to larger receptive field of the OP generator than those of the a‐ and b‐waves, respectively, concurring with the spatial summation of the oscillatory response having been found to be wider than that of the a‐ and b‐waves and also than that of the amacrine cells (proximal negative response) in the amphibian retina (Hahn & Wachtmeister, 1989; Wachtmeister & Hahn, 1987). In addition, as mentioned, some impact of retinal remodelling after progressive loss of photoreceptors cannot be excluded, all depending on different stages and severities of the disease which can be expressed in the altered oscillatory response.

Furthermore, it has been reported that the cones and their retinal pathways may remain functional even as degeneration is progressing and that the so‐called ‘dormant’ cones have been observed in the mice retina (Ellis et al., 2023) and may thus be reflected in the preserved oscillatory response.

In short, also in RP, the oscillatory response of the ffERG as well as of the fmERG indicates neuronal dysfunction of the mixed rod–cone pathway of the inner retina in a relatively, objectively and sensitively way reflecting the different states and locations and progression of the RP disease.

More detailed descriptions of the oscillatory response and individual oscillatory peaks in fleck diseases including cone–rod (i.e. Stargartds/Fundus flavimaculatus) and rod–cone (i.e. Fundus albipunctata) have previously been reported in surveys and will be referred to (Wachtmeister, 1998, 2001).

#### 5.1.2 Acquired (neuronal) retinal diseases

##### Epiretinal membranes/macular holes (pseudo‐holes, penetrating holes)

The oscillatory response of the fmERG—5 and 15°, respectively—in patients (*n* = 22) with epiretinal membranes (ERM) and pseudoholes in the macula has been analysed by Suzuki et al. (2003). A significant reduction in amplitudes and timing of all components—the OPs having the largest decrease in the fmERG (15°), and a significant delay of OP1, was observed. Thus, an abnormal oscillatory response seems to most sensitively signal malfunction of the neuronal pathway in parafoveal inner retina in ERM with pseudohole.

The OPs of the mfERG have been employed to examine the effect of removing the inner limiting membrane (ILM) at successful surgery for full thickness penetrating macular hole in 10 patients (Ogata et al., 2007). The visual acuity was improved, while no significant difference between the pre‐ and postoperative oscillatory response, neither between the area with removed ILM (20°) and the outside area (40°), was recorded. The outcome of a macular hole surgery technique, including ILM removal, could thus be evaluated to do no harm to the neuronal function in the inner retina of the posterior pole, expressed in the oscillatory response.

The oscillatory response of the fmERG has been used (15 patients, 16 eyes) to evaluate functional changes in the inner and middle layer of the parafoveal (15°) retina after removal of the ERM by Hibi et al. (2013). The OPs (OP1, OP2, OP3), preoperatively reduced, increased (1.6 times) after surgery, but did not recover to normal range. There was a higher correlation for the OPs than that for the a‐ and b‐waves to the middle (OPL, INL, IPL, GC) retinal thickness measured with SD‐OCT (Optical Coherence Tomography).

Thus, in cases in need for macular surgical intervention, the OPs may sensitively and selectively reflect the postoperative functional status of the changed morphology in the inner/middle retina in early stages of ERM before the progression and damage of the outer retina.

##### Myopia

Chen et al. (2006) analysed the oscillatory response of the mfERG in myopia (*n* = 18) to differentiate between stationary and progressive types. In both forms, as well as in emmetropes, OP2 and OP3 increased with eccentricity and were more prominent parafoveally (17–20°) than in the foveal area (0–9°). The temporal OPs were also significantly greater than in the nasal hemifields. Myopes with progressive form had OPs with significantly shorter implicit times in all eccentric areas compared to the stable type as well as to emmetropes.

In 2020, Wan et al. (2020) studied the oscillatory response of the rod‐ and cone‐driven ffERG in different degrees of myopia (*n* = 57) using wavelet Fourier to analyse the frequency spectrum of the OPs. A significant (positive) correlation between the refractive power and average peak frequency and to the amplitude of the extracted rod‐driven OPs was observed, the peak frequency being higher in myopes than in emmetropes. The light‐adapted cone‐driven OPs were within normal range.

Thus, the OPs of the mfERG may valuably assist in the differentiation between stationary and progressive types of myopia, and further, the oscillatory response of extracted rod‐driven ffERG seems to reflect the effect by different degrees of myopia on neuronal function in the retina.

##### Melanoma‐associated retinopathy (MAR), central serous chorioretinopathy (CS(C)R)

In a case of melanoma‐associated retinopathy (MAR), a selective absence of the oscillatory response and a reduced b‐wave in the dark‐adapted rod ffERG as well as a severely reduced ON‐component have been reported (Alexander et al., 1992).

Already in 1988, Miyake et al. (1988) described the OPs of the fmERG to be reduced with prolonged implicit times in central serous chorioretinopathy (CS(C)R) and more severely diminished than the a‐ and b‐waves. Post‐treatment the a‐ and b‐waves recovered to control level, but not the oscillatory response.

Thus, monitoring of the OPs of the ffERG and fmERG may be relatively more sensitive indicators of the presence of acquired retinal neuronal disease, that is, in MAR and post‐therapeutic remaining neuronal dysfunction in the inner retina in, that is, CS(C)R.

### 5.2 Vascular diseases

Vascular diseases of the retina can significantly alter the OPs, reflecting disruption of the retinal microcirculation and inner retinal function. This section highlights OP changes in conditions such as diabetic and hypertensive retinopathy, vascular occlusions and retinopathy of prematurity.

#### 5.2.1 Diabetic retinopathy

Diabetic retinopathy (DR) is clinically classified as a vascular disease and usually graded according to appearance and extent of pathological vascular findings in the ocular fundus.

However, since the early clinical study of Simonsen (1965) using ffERG recordings including evaluation of the oscillatory response, the OPs have been reported to be abnormal also at an early stage of diabetes without visible retinal microvascular changes and to indicate neuronal dysfunction. Many studies followed, clinical as well as experimental, using more modern techniques like fmERG, mfERG and pattern ERG indicating the presence of neural dysfunction in DR, and several important reviews (Bearse et al., 2006; McAnany et al., 2022; Tzekov & Arden, 1999) strongly support this view. Recently, Lynch and Abramoff (2017) referring to clinical and experimental data even concluded that the diabetic retinal neurodegeneration (DRN) may precede the earliest manifestation of DR.

The importance of retinal hypoxia, increasing during hours of darkness, has also been highlighted to exist in the earliest stage of diabetes and involving cytokines, specifically reactive oxygen species (ROS) (Arden & Sivaprasad, 2012). The oscillatory response in diabetic patients has also been observed to be affected by inhalation of oxygen (Drasdo et al., 2002).

The relationship between the OPs of the ffERG and vascular calibre in patients (*n* = 24) with type 2 diabetes with no diabetes retinopathy (no DR, *n* = 14) and non‐proliferative retinopathy (NPDR, *n* = 10) was studied by Luu et al. (2010). All the OPs (OP1‐4) were significantly reduced and delayed, and there was no difference between the two groups (nDR and NPDR). The amplitude of OP4 correlated significantly with the calibre of the arterioles. Thus, in the early stages of diabetes, there seems to be a substantial vascular retinal dysfunction reflected in a reduced oscillatory response, and OP4 was observed to be predominantly affected and correlating with the arteriolar calibre in the diabetic retina.

Midena et al. (2021) studying the alterations of the OPs of the ffERG and early vascular parameters in patients (*n* = 22) without or mild DR described significant reductions of the amplitudes of OP2, 3 and 4 and the sum of the OPs. A direct correlation between the OP response and vessel lengths and fraction dimensions in the superior vascular plexus was noted. An inverse correlation between the OPs and vessel area density in the inner and deeper vascular plexus in the retina was described. The relevance of ‘crosstalk’ between the retinal neurons, most probably the amacrines, and the vascular components in the diabetic retina was emphasized.

Therefore, there seems still to be only circumstantial and not conclusive evidence if DR will be a primary vasculopathy or neuropathy.

Holopigian et al. (1990) studied the OPs of the ffERG in diabetics (4 type 1 and 10 type 2) under both photopic and scotopic conditions. A significant, uniform loss (50%) of the scotopic OP amplitudes was demonstrated. An increase in implicit time of the scotopic b‐wave but no delay of the OPs was observed. There was also an abnormal flicker response. They concluded that both the scotopic and photopic systems show losses in early diabetic retinopathy indicating that lesions besides the inner plexiform layer with bipolar cells are affected but additional retinal sites in the mid‐retinal levels reflected in the abnormal oscillatory response.

A delay of the first peak OP1 and an overall reduction of the oscillatory response of the ffERG have been reported in several studies to be present and to be a consistent alteration in patients with no apparent DR (stage O), see review (Tzekov & Arden, 1999).

There are also experimental studies (Glass et al., 2023; Hancock & Kraft, 2004) showing reduced and delayed amplitudes of the OPs of the ffERG (rats) as well as morphological changes—a thinner retina—reduced bipolars and Muller cells in diabetic retinas (mice).

In 2020, the relation between the oscillatory response to cone phototransduction (activation phase) of the photopic ffERG in 40 diabetic patients, 20 without and 20 with mild DR was investigated by McAnany et al. (2020). Log OP amplitudes were significantly reduced as well as the phototransduction sensitivity, measured as a mathematical derivation of the a‐wave response. The later OPs, OP3 and 4, tended to be more reduced than the earlier ones. However, no significant correlation between the cone sensitivity and the OPs were observed.

Thus, the loss of the OPs of the ffERG in DR, most often recorded under DA conditions, also found under photopic conditions, being independent of the cone photoreceptor sensitivity and thus directly on photoreceptor function, is also well supported by the literature (see also reviews McAnany et al., 2022; Tzekov & Arden, 1999; Wachtmeister, 1998). In sum, the OP response of the ffERG is comparatively more sensitive than pattern ERG (Arden et al., 1986), correlates better to the grade of retinopathy than the scotopic threshold response (Aylward, 1989), and the a‐ and b‐waves are normal unless the disease is very advanced. The usefulness of measurement, analysis and monitoring the oscillatory response of the ffERG for diagnosis and prediction of progress of DR disease will be found more in detail in the reviews (McAnany et al., 2022; Tzekov & Arden, 1999; Wachtmeister, 1998).

In 2004, the oscillatory response of the slow flash multifocal ERG (sfmf ERG) was studied in 16 diabetic subjects without retinopathy (noR) and 16 with early non‐proliferative RP (NPRP) by Bearse et al. (2004). Local mfOPs were isolated by using a special technique to minimize noise level (SNR) for analysis of the first‐order (K1), second‐order (K2), etc., components for each retinal location of the central 45 degrees of the fundus. Abnormal, in either component of the mfOPs, were found in 19%–25% in noR eyes and in both components in 62% in NPDR eyes. One mfOP component (Ks2mf) representing a fast adaptational mechanism was more associated with NPDR than another (K1) component. They conclude that adaptation is more likely to be abnormal in early diabetic retinopathy reflected in the oscillatory response and may precede the development of DR and have a stronger power for prediction.

The mfOPs of nine subjects with diabetes 1 with no overt (one with 2 microaneurysms) retinopathy were examined to explore the effect of compromised microvascular perfusion by increasing oxygen supply inhaling carbon (Kurtenbach et al., 2010). The first‐order kernel (OPs 1–4) and the second‐order kernel (OPs 1–3) responses in the central 60° retina were analysed. In controls, but not in diabetics, a significant alteration (increase) of second‐order OP3 amplitude topography was recorded. Carbon inhalation also induced a small, but not significant increase of the first‐order OP2 in both groups. Thus, the oscillatory response seems to reflect a sensitivity to carbon inhalation with compromised vasculature in diabetics.

In short, recording of the mfOPs to evaluate the function of the central part of fundus seems to contribute to a better understanding of the pathophysiology of the diabetic retina reflecting both the neurological and vascular components of the disease.

#### 5.2.2 Hypertensive retinopathy

Bellini et al. (1995) described that the OP index (OPI), sum of OP1, OP2 and OP3 of the ffERG to be significantly reduced in mild and moderate essential hypertension (*n* = 24, 28–50 years old) before the onset of morphological changes specific for hypertensive retinopathy. The systolic blood pressure appeared strongly influencing the OPI. The OPI has also been successfully used to evaluate the therapeutic effect in hypertension (Ravalico et al., 1995).

Experimental studies of the OPs of the ffERG have been performed. Reichardt and Fischer (2016) used transgenic rats (dTGR) with elevated angiotensin II and severe hypertension. Structural alterations with degeneration of the ganglion cells, alterations in the outer blood‐retinal barrier and mild functional loss of both rod and cone function, reflected in last but not least a reduced oscillatory response.

Thus, a reduced oscillatory response may also be presented in mild hypertension indicating structural alteration in the inner retinal layers and should be of value in clinical trials of therapy.

#### 5.2.3 Vascular occlusions

##### Arterial occlusions

There are limited studies per se on the pathophysiology and dysfunction of the retinal artery occlusion‐central or branch. Lee et al. (Lee, Jeong, et al., 2021) in their experimental investigation on the ocular ischaemic syndrome (OIS) used a murine model with unilateral carotid artery occlusion to induce retinal ischaemia in mice. Ocular dysfunction was evaluated by analysing the ffERG including the oscillatory response. Impaired OPs were constantly observed as well as Muller cell gliosis and inflammation and loss of ganglion cells. The OPs have also been used to measure the outcome of treatment with a neuroprotective drug (pemafibrate) for retinal ischaemia using the same murine model (Lee, Tomita, et al., 2021). The oscillatory response was described to be preserved and the retinal gliosis modulated.

Thus, in arterial retinal occlusion, dramatic functional impairment in the inner retina occurs as reflected in a constantly reduced oscillatory response, which also may be used to sensitively measure the outcome of clinical trials.

##### Venous occlusions

Already in 1994, Hara and Miura (1994) reported in patients (*n* = 34) with branch retinal vein occlusion (BRVO) that the OPs (OP1‐OP4) of the ffERG to be significantly reduced, although none of the conventional ERG variables were significantly changed. They concluded that the oscillatory response to be a most sensitive indicator for BRVO and damage to the inner retina.

A novel more advanced method to analyse the oscillatory response of the ffERG in patients (*n* = 17) with central retinal vein occlusion (CRVO) and healthy eyes has been presented by Sefandarmaz et al. (2020). Techniques were used like parabolic mapping, density criteria, etc., and statistical differences were reported between the CRVO and healthy eyes, and the severity of the disease was also possible to estimate.

More recently, the oscillatory response of the ffERG was studied in patients with first episode acute unilateral CRVO—11 eyes with ischaemic type and 32 eyes with non‐ischaemic type—identified by fundus fluorescein angiography (FFA) (Qu et al., 2024). The individual OPs (OP1, OP2, OP3, but not OP4) and the sum of OPs in the CRVO eyes were significantly reduced to 40% of the values of the unaffected control eyes. The oscillatory response (sum of OP1–OP3) in the ischaemic eye group was significantly different compared to the non‐ischaemic eye group, but there was no peak delay. Thus, the OPs of the ffERG seem to be a sensitive and quantitative indicator to be used for functional assessment of the retina and the severity of the CRVO disease.

Nishimura et al. (2020) evaluated the OPs of the fmERG and ffERG in 20 patients with non‐ischaemic CRVO in a clinical trial of treat‐and‐extend (TAE) intravitreal afibercept (IVA), an anti‐vascular endothelial growth factor (anti‐VGF). The OPs of the fmERG and ffERG were reported to be affected during the 12‐month follow‐up and did not have a recovery, despite the macula oedema and thickness were reduced.

Thus, the oscillatory response may assist in assessment of diagnosis and objectively measure the outcome of retinal treatment. Furthermore, in non‐ischaemic CRVO, the OPs will indicate a permanent damage in the inner retina and specifically the amacrines and probably the ganglion cells.

#### 5.2.4 Retinopathy of prematurity (ROP)

In 2007, Akula et al. (2007) in their study of the OPs of the ffERG in two rat models of ROP also characterized human subjects with a history of mild ROP. Human adults with a history of ROP (*n* = 14) had lower energy OP than control adults, but ROP infants had higher energy OPs than control infants. The dominant frequency and sensitivity of ROP subjects compared to healthy subjects did not change even though the sensitivity in adults was greater than that in infants.

The dark‐adapted OPs of the ffERG in premature infants (*n* = 28) with and without ROP were studied by Mactier et al. (2013). Infants at threshold ROP presented the summed amplitudes of (OP1, OP2, etc.) to be smaller, also at 50 weeks postnatal age. OP1 was less likely to be present in the infants who developed stage 3 and worse ROP. The prevalence of the later OPs (OP2. OP3, OP4, OP5) did not differ.

Thus, the OPs measure retinal sensitivity in ROP in a way not specified by the a‐ and b‐waves to provide additional insight in the disease process in the inner neural retina in ROP.

### 5.3 Mixed neuronal/vascular diseases

This part includes pathological conditions that are regarded as neuronal diseases per se, that is, primarily degeneration of the optic nerve, the optic nerve head, the retinal nerve fibre layer (RNFL) and the ganglion cells (RGC) but may also involve comprised microvascular network in the highly structured retina.

#### 5.3.1 Inherited optic neuropathy

##### Leber's hereditary optic neuropathy (LHON)

In the rare Leber's hereditary optic neuropathy (LHON) having preserved photoreceptor and bipolar functions (but dysfunction of the ganglion cells, reflected in a pathological pattern ERG), Barboni et al. (2024) recently reported reduced OPs of the ffERG, mainly affecting the OP2, in young adult patients (*n* = 7). The photopic negative response (PhNR) was also reduced but not correlated to the OP response.

Using mfERG (second kernel, 1st slice), Kurtenbach et al. (2004) described inner retinal dysfunction in patients (*n* = 9) with LHON. In addition, with careful observation of their recordings, alterations in the wavelet responses seem to be present.

Thus, the OPs seem to be reduced early in the disease process reflecting retinal dysfunction in presymptomatic stages of LHON, later affecting the RGC and indicative of the proximity and physiological interdependence of the RGCs to the OP generator(s).

##### Autosomal dominant optic atrophy

Relatively old patients (*n* = 8) with autosomal dominant optic atrophy (ADOA), the most common form of hereditary optic neuropathy, have been reported by Miyata et al. (2007) to have severely reduced OPs of the ffERG. The reduction of the OPs could not be explained by an overall ERG decrease, as the mean ratio OP/b‐wave was significantly lower. There was also an inverse correlation with age indicating the dysfunction of retinal neuronal circuits in the inner retina to give rise to the OPs being secondary to the ganglion cell degeneration.

To summarize, reduced OPs seem to signal early presymptomatic hereditary optic neuropathy in, that is, LHON and in the most common hereditary one, that is, ADOA the oscillatory response is severely affected, secondary to ganglion cell degeneration.

#### 5.3.2 Glaucoma

Already in 1987, Gur et al. (1987) studying the dark‐adapted and progressive light‐adapted ffERG in glaucoma patients (*n* = 45) reported that the glaucomatous lesion may extend more distally than the ganglion cell layer. Using special recording conditions (inter‐flash interval for estimating maximal amplitude) and measuring the root mean square (RMS) of the amplitudes of the oscillatory response, abnormal changes in one or both indices were noted in most of the glaucoma patients.

Later Fortune et al. (2002) applied a specific mfERG paradigm (a scaled down photopic ffERG)—using an m‐sequence cycle with interposing separate focal, flashes stimuli and intervening blank frames. An analysis was made of the first‐order kernel response with the direct component (DC)—similar to the dim photopic response—followed by the induced component (IC) of the central macula. Focus was concentrated on the distinct oscillation at 70 msec in the temporal retina representing an adaptive effect within retina within a train of stimulus responses. In patients (*n* = 16) with primary open angle glaucoma (POAG), a selective loss of the oscillatory part (from the temporal retina) in the IC‐response was observed. The latency of this temporal oscillation was also slightly prolonged.

Thus, in glaucoma, the oscillatory component may sensitively indicate disturbed inner retinal processing besides pathology of the optic nerve head and ganglion cells. More specifically, an abnormality in the inner plexiform layer and diminished adaptive ‘gain control’ from distal to proximal layers may occur early in POAG disease.

Interestingly and more recently, Yoshikawa et al. (2022) studied the retinal function and vascular structure in patients (*n* = 24) with glaucoma. A significant positive correlation of the P1‐N1 of the mfERG and the macular deep vessel density evaluated with optical coherence tomography was observed. Thus, deep retinal layers dysfunction involving ischaemic bipolar and amacrine cells seems to be associated with glaucoma and be reflected by an abnormal oscillatory response as described above.

There are also animal studies of retinal function in glaucoma. In a primate model, the slow sequence of mfERG was used focusing on the frequency(ies) of the oscillatory response (Rangaswamy et al., 2006). In adult monkeys (*n* = 8) laser‐induced glaucoma, the high‐frequency OPs (approximately peak at 145 Hz) were reduced in amplitude, also with diminished naso‐temporal asymmetry. The slow OPs (peak frequency around 77 Hz) were less affected. The summed fast OPs amplitudes (root mean square, RMS) were also observed to better correlate to the MD visual field (difference in sensitivity over the entire retina in perimetric testing of the animals). Thus, the fast OPs, relying more on the integrity of the ganglion cells and their axons than the slow ones, were described to be preferable for monitoring the progression of the glaucoma disease.

In summary and briefly, the glaucoma disease is complex and known experimentally, that is, in mouse (Lakshmanan et al., 2023) to reduce retinal function, gradual loss of nerve fibre and ganglion cell, combined or primarily, with vascular pathology involving the inner retinal layers. A finding of an abnormal oscillatory response reflecting a compromised inner retinal function may thus assist in diagnosis, follow the progression of glaucoma and evaluate outcome of therapeutic trials.

### 5.4 Acquired/toxic retinal disorders/miscellaneous

#### 5.4.1 Vitamin A deficiency

Recently, retinal electrophysiology results (ffERG and mfERG) in a case of Vitamin A deficiency (VAD) retinopathy due to coeliac disease and liver fibrosis have been reported by Pereira et al. (2024). Even though no detailed analysis of the oscillatory response was performed, a scrutinized inspection of the ffERG recordings presented in figure 2 in Pereira et al. (2024) shows smaller wavelets compared to the age‐matched control—also after Vitamin A replacement therapy with 6‐month follow‐up.

In an experimental study of early stage VAD in rats, the OPs of the ffERG have been reported to be significantly reduced and more greatly affected than the a‐ and b‐waves (Kakiuchi et al., 2015). The mean amplitudes of OP1, OP2 and OP3 and the sum of the OPs (OP1‐OP4) in VAD rats were recorded to be significantly decreased (35%–71%) compared to the controls. After a 5‐week recovery period, the functional impairment returned to normal.

Thus, in VAD, reduction of the oscillatory response sensitively indicates that not only the function of photoreceptors is affected but also the signal transduction in the inner retinal layers via probably the amacrines and their transmitters including GABA, glycine and dopamine.

#### 5.4.2 Nicotine

The effects of nicotine on retinal function in healthy visually normal adults (*n* = 10) non‐smokers were studied by Varghese et al. (2011). Representative recordings of OP waveforms of the ffERG in DA and LA after oral intake (gum) of nicotine (2 and 4 mg) were shown and described. There were no differences in the summed OPs across the conditions. The OP results were presented in the discussion section and reported to be an increase in the OP2 amplitude in the DA condition and augmentations of OP2, 3 and 5, respectively, in the LA ERG. Thus, there seems to be indications of a selective change in the individual OPs by nicotine, but no conclusive cause and effect.

#### 5.4.3 Autism

In autism spectrum disorder, that has been observed to have signs of earlier ageing in CNS including retina, the oscillatory response of the ffERG has been reported to be different (Constable et al., 2016). An earlier bifurcation of the LA OP2, that is normally seen in more aged patients, was observed in the younger participants (*n* = 11). There was no difference in the a‐wave and the phNR, but a reduced b‐wave at the ON‐set of the LA ERG. Thus, extensive and thorough analysis detecting the very different waveform of the oscillatory response may also be used as a clinical measure of CNS function in this neurodevelopmental disorder.

To conclude this section (5th), there is a vast number of clinical conditions that, earlier and/or later, have an impact on the function of the inner retinal layer as reflected in a change of the oscillatory response. Early signs of vascular and/or neuronal diseases may be indicated by altered amplitudes or timing of all or individual wavelets of the ffERG, mfERG or fmERG. Further studies are needed to relate the impaired oscillatory response and rapid neuronal adaptation to psychophysical data. The loss of this capacity of the visual system—the neuronal adaptive activity—is indeed most significant in a human as well as in animals. A rapid response to change from darkness to light or vice versa—an important feature and necessary for survival.

## 6 CONCLUSIONS

The present survey has made an effort to provide current knowledge about the oscillatory response and its relation to neuronal adaptation.

The precise and cellular origin(s) of the OPs is still a challenge. The early and late peaks have different origins. The short latency OPs seem to be rod‐driven and the later ones cone‐driven. The photopic OPs originate predominately from the ON pathways. The very first wavelet may be coupled to the a‐wave and be photoreceptor‐driven. The later OPs are associated with the OFF‐bipolar cells. The oscillatory response represents activity in inhibitory, probably dopaminergic‐GABAergic neuronal feedback pathways in the inner plexiform layer, initiated by glycinergic type of amacrine cells—tentatively AII‐type.

For clinical and research applications, OP analysis can serve as a marker of neuronal (network), non‐photochemical, swiftly working adaptive capacity of the retina. Key indicators include amplitude or energy changes (OP1–OP4), latency shifts and frequency alterations under controlled adaptational states. New techniques and forms of analyses like recording of the mfOPs to calculate frequencies, spectra and measuring scotopic ffOPs to very dim flashes have evolved and seem to have improved the possibility for earlier detection and diagnosis of retinal disease.

Analysis of the OPs reflecting neuronal adaptive capacity is relevant for several retinal diseases, mainly vascular and neuro‐degenerative disorders, that is, diabetic retinopathy and retinal dystrophies, assisting in diagnosis, follow‐up and evaluation of the effect of retinal treatment. The assessment of the wavelets seems also to be of value to separate different genotypes and phenotypes in hereditary retinal degenerations and to judge outcomes in clinical trials for future therapy of retinal dysfunction. Therefore, recording of OPs has a role in clinical ophthalmology for testing retinal function in health and disease.

To conclude, the oscillatory response reflects the complex activity in the neural microcircuitry of the inner retina ‐ the neuronal adaptation. Its most vital function, in all mammals including man, is to quickly adapt and adjust vision from dark to light and vice versa.
